# Multiparametric CMR for detection of cardiac amyloidosis in patients with undifferentiated concentric left ventricular hypertrophy

**DOI:** 10.1007/s10554-026-03616-w

**Published:** 2026-02-02

**Authors:** Sandra Quinn, Andrew Zbihley, Umar Ramzan, Connor Raikar, Joshua Engel, James C. Carr, Bradley D. Allen

**Affiliations:** 1 Department of Radiology, Northwestern University Feinberg School of Medicine, Chicago, IL USA; 2 Department of Cardiology, St James’s Hospital, Dublin, Ireland

## Abstract

Multiparametric CMR can detect cardiac amyloidosis (CA) in patients with undifferentiated concentric left ventricular hypertrophy (LVH). A retrospective patient search was performed for patients ≥ 18 years of age who received 1.5T CMR from January 2018 to August 2022 to evaluate for myocardial infiltration in the setting of known concentric LVH at a tertiary medical center. Clinical records were reviewed for positive diagnosis or exclusion of CA. CMR were post-processed to evaluate ventricular volumes and function, native T1 mapping, and extracellular volume (ECV). Late gadolinium enhancement (LGE) was qualitatively evaluated and each segment categorized into vascular or non-vascular LGE using the American Heart Association 16 segment model. Feature tracking strain (FTS) was performed on a subset of CA positive and CA negative patients. Group comparisons were made using one-way ANOVA (parametric) or Kruskal-Wallis (non-parametric) tests. Receiver operator characteristic (ROC) analysis with area under curve (AUC) values were generated for both individual and combined parameters using binary logistic regression (IBM SPSS Statistics V26.0, and DATATab 2025) to determine optimal cut-off parameters for detection of CA. CMR were performed in 278 patients for myocardial infiltration evaluation in the setting of known concentric LVH (mean age 63.2 ± 14.9, 46% female). Diagnostic groups were determined as follows: CA positive (*n* = 60), CA negative (*n* = 100) and CA unknown (*n* = 118). CA positive patients, when compared to both CA negative and CA unknown groups, respectively, demonstrated significantly higher age (69.9 ± 10.3 vs. 59.7 ± 14.2 and 62.9 ± 16.4 years), native T1 (1122.4 ± 64.6 vs. 1056.8 ± 69.7 and 1051.4 ± 54.0 ms), ECV (46.4 ± 11.5 vs. 32.1 ± 7.2 and 32.1 ± 7.6%) and number of segments with infarct-atypical LGE (10.2 ± 7.3 vs. 2.7 ± 4.7 and 2.0 ± 4.3). ROC AUC values were calculated for native T1 (0.80), ECV (0.88), and number of infarct-atypical LGE segments (0.76). A 4 parameter model including age, native T1, ECV, number of segments with non-vascular LGE demonstrated an AUC of 0.91 for detection of CA, with a sensitivity of 92% and specificity of 81%, which when applied to the CA unknown group, indicated 13 patients (11%) in this group may have CA. CA positive patients demonstrated reduced basal peak systolic strain and diastolic strain rates when compared to CA negative patients; a model combining these parameters with patient age demonstrated an AUC of 0.79 for detection of CA. Multiparametric CMR can discriminate CA positive patients from CA negative and undifferentiated LVH patients in a real-world tertiary center population. These findings demonstrate that CMR has significant diagnostic potential for detection of CA in patients with undifferentiated LVH.

## Introduction

Cardiac amyloidosis (CA) has become increasingly recognized as the underlying pathology in patients with unexplained left ventricular hypertrophy (LVH) or heart failure with preserved ejection fraction [1]. The majority of CA cases occur due to either amyloid immunoglobulin light chain (AL), a monoclonal light chain produced by plasma cells, or amyloid transthyretin (ATTR), a thyroid hormone transport protein produced by the liver. The AL or ATTR misfolded fibrils gradually deposit and accumulate in the extracellular matrix of the myocardium resulting in myocardial thickening. The subsequent infiltrative cardiomyopathy that evolves is characterized by elevated left ventricular filling pressures, restrictive physiology and then finally progression to the clinical syndrome of heart failure [1]. Additionally, disruption of the cardiac conduction system as a result of these progressive amyloid fibril deposits can lead to symptomatic bradyarrhythmia requiring a permanent pacemaker. Furthermore, ventricular tachycardia and sudden cardiac death are also recognized risks of CA [2].

Clinical management of patients with CA has historically been focused predominantly on maintaining a state of clinical decongestion and management of arrhythmia, however in recent times the advent of disease modifying therapies for ATTR-CA has spurred the medical and scientific communities to seek strategies to identify these patients earlier in their clinical course. Although contemporary non-invasive diagnostic criteria exist for simplifying the process for ATTR CA diagnosis [3, 4], CA overall remains significantly under-recognised in real-world clinical practice [1, 5, 6]. With a significant morbidity and mortality burden associated with CA [7, 8], identification of this condition earlier in the disease process and avoidance of diagnostic delay may prove prudent for improving outcomes in this patient group.

Standard cardiac magnetic resonance (CMR) parameters have proven their utility in the identification of structural abnormalities and cardiac remodelling suggestive of CA. Specifically, the ability of CMR to perform myocardial tissue characterisation with native T1 mapping, late gadolinium enhancement (LGE) and extracellular volume (ECV) gives CMR a distinct advantage over other imaging modalities in the context of CA. There is anticipation that myocardial tissue characterisation may provide a quantitative measure to diagnose CA and track response to treatment over time, and feature tracking strain (FTS) may provide more understanding of cardiac function and myocardial deformation in CA patients [9–11].

The aim of this study was to evaluate the utility of CMR in distinguishing CA from other causes of concentric LVH in a real-world cohort of patients with undifferentiated LVH.

## Methods

### Study population

This study was approved by the institutional review board and conducted using a retrospective design; therefore, the requirement for informed consent was waived. Patients ≥ 18 years of age were retrospectively identified as having undergone clinically-requested 1.5T CMR for investigation for cause of left ventricular hypertrophy, heart failure preserved ejection fraction, or infiltrative disease at our center between Jan 2018 and Aug 2022. Patients were included who had confirmed concentric LVH detected with echocardiography prior to the CMR, defined as a relative wall thickness (RWT) > 0.42 and LV mass index > 95 g/m^2^ (female) or 115 g/m^2^ (male). Patients were stratified into 3 groups; those who were subsequently diagnosed with CA (“CA positive”), those where a CA diagnosis was excluded (“CA negative”) and all other patients without confirmed or excluded CA with no clear alternative diagnosis (“CA unknown”). Diagnosis of CA was defined as follows [12]:

(i) Endomyocardial biopsy consistent with CA in conjunction with typical echocardiographic findings.

Or, in the absence of cardiac biopsy:

(ii) ATTR CA diagnosis required all of the following:
  - Grade 2 or 3 cardiac uptake of ^99m^Tc-PYP with cardiac scintigraphy confirmed with SPECT imaging.
  - Absence of evidence of monoclonal paraprotein protein as determined by negative serum free light chain analysis and negative blood serum or urine immunofixation.
  - Typical echocardiographic or CMR features of CA.
(iii) AL CA diagnosis required all of the following:

- Evidence of monoclonal protein with serum free light chain analysis or positive finding of monoclonal protein on blood or urine immunofixation.
- Non-cardiac biopsy consistent with systemic AL amyloidosis.
- Typical echocardiographic or CMR features of CA.

Typical echocardiographic features of CA were defined as any of the following [12]:

(i) Left ventricular wall thickness > 12 mm.
(ii) ≥ Grade 2 diastolic dysfunction.
(iii) Relative apical sparing of global longitudinal strain ratio (i.e. the average apical LS/average combined mid and basal LS > 1).

### CMR acquisition

Clinical CMR examinations were performed using a 1.5-Tesla MRI system (MAGNETOM Aera or Avanto, Siemens Healthineers, Germany). The CMR protocol included cardiac function and tissue parameters, assessed by 2D balanced steady-state free precession (bSSFP) cine imaging, pre- and post-gadolinium T1 mapping, T2 mapping, and LGE phase sensitive inversion recovery (PSIR) and/or magnitude-only inversion recovery (MAG) sequences. Cine balanced steady-state free precession (bSSFP) sequences were employed to image the ventricles in standard cardiac planes, including short-axis views (comprising 8–12 contiguous slices) and long-axis orientations (2-, 3-, and 4-chamber). The short-axis stack extended from the ventricular base to the apex and was reconstructed into 25 cardiac phases. Imaging parameters included a repetition time (TR) of 2.6–3.0 ms, echo time (TE) of 1.0–1.5 ms, flip angle between 45° and 70°, and a bandwidth per pixel of 700–1000 Hz. GRAPPA acceleration with a factor of *R* = 2 was applied. Spatial resolution ranged from 1.5 to 2.3 mm² in-plane, with a slice thickness of 6–8 mm. T1 and T2 mapping sequences were performed at three short-axis levels (basal, mid-ventricular, and apical) during breath-holds. Native and post-contrast T1 mapping was acquired using a modified Look-Locker inversion recovery (MOLLI) technique. Native T1 mapping used a 5(3)3 sampling scheme, while post-contrast T1 (acquired 15 minutes after 8 administration of gadolinium contrast agent [Gadavist, 0.1 mmol/kg; Bayer, Leverkusen, Germany]) used a 4(1)3(1)2 scheme, both over 11 cardiac cycles. Imaging parameters included a TE of 1.0–1.3 ms, TR of 2.5–4.2 ms, in-plane resolution of 1.0–2.1 mm × 1.5–2.5 mm, slice thickness of 8 mm, and flip angle of 35°. Inline motion correction was applied, and parametric T1 maps were generated automatically. LGE imaging was performed 10–15 min after intravenous administration of gadobutrol (Gadavist, 0.1 mmol/kg; Bayer, Leverkusen, Germany) using an inversion-recovery-prepared gradient echo sequence. Images were acquired in standard short-axis and long-axis stacks covering the full left ventricle. PSIR reconstruction was used to facilitate accurate nulling of normal myocardium and to improve contrast between healthy and fibrotic tissue. Both MAG and PSIR images were reconstructed and stored. The inversion time (TI) was individually adjusted using a TI-scout sequence to null normal myocardium. Imaging parameters included a TE of 3.0–4.0 ms, TR of 7.0–9.0 ms, flip angle of 25–30°, in-plane resolution of 1.3–2.0 mm × 1.3–2.0 mm, and slice thickness of 6–8 mm.

### CMR post-processing

Cardiac image analysis was performed using dedicated post-processing software (cvi42, version 5.13, Circle Cardiovascular Imaging, Calgary, Canada). Biventricular volumetric assessment was conducted by manual delineation of endocardial and epicardial borders at both end-diastole and end-systole across the short-axis cine stack. Papillary muscles and trabeculations were excluded from the blood pool. Left and right ventricular end-diastolic volume (EDV), end-systolic volume (ESV), stroke volume (SV), and ejection fraction (EF) were calculated, and all volumetric parameters were indexed to body surface area (BSA). T1 and T2 parametric maps were analyzed using the same software platform. Native T1, post-contrast T1, and T2 values were quantified from myocardium within manually drawn endocardial and epicardial borders with 15% offset at base, mid and apical slices, with measurements averaged across all segments according to the 16-segment American Heart Association (AHA) model. For ECV estimation, blood pool regions of interest (ROIs) were drawn manually on both native and post-contrast T1 maps in the mid-ventricular slice. ECV was calculated using the standard formula: ECV = (Δ(1/T1_myocardium)/Δ(1/T1_bloodpool)) × (1 – hematocrit), with hematocrit values obtained from blood sampling. LGE characterisation was determined by a single experienced observer and classified as ‘infarct atypical’ (i.e. midmyocardial, subepicardial, or diffuse subendocardial in a non-vascular territorial distribution) or ‘infarct typical’ (i.e. subendocardial or transmural LGE, well-delineated, conforms to a vascular distribution). In a subset of CA positive and CA negative patients, 2D feature tracking strain analysis was performed using the short-axis cine stack and 2-, 3- and 4-chamber views. Endocardial and epicardial borders were manually contoured at end-diastole, and myocardial motion was automatically tracked throughout the cardiac cycle to calculate global and regional strain parameters. Feature-tracking strain (FTS) analysis was performed in patients with cine sequences available in a format compatible with post-processing; acquisitions were excluded if there was a mismatch in phase count across all views or if observer opinion deemed endocardial border tracking unreliable.

### Statistical analysis

Variables were grouped for statistical analysis as follows based on diagnosis or exclusion of cardiac amyloidosis (CA): (i) CA positive, (ii) CA negative, and (iii) CA unknown. Descriptive statistics were used to present continuous variables as mean ± standard deviation. Group comparisons were made using one-way ANOVA for parametric data or the Kruskal-Wallis test for non-parametric data, with Bonferroni correction applied to post-hoc pairwise comparisons where appropriate. Categorical variables were compared using the Chi-square test. Receiver operator characteristic (ROC) analysis with area under curve (AUC) values were generated for both individual and combined parameters using binary logistic regression, and optimal cut-off values for detection of CA determined using Youden’s index. The logistic regression model determined from the CA positive and CA negative groups for detection of CA was applied to the CA unknown group, to identify patient’s who potentially may have CA. Statistical analyses were conducted using SPSS Statistics software (V26.0, IBM Corp, Armonk, NY, USA), and DATATab 2025, with *P* < 0.05 indicating statistical significance.

## Results

### Baseline characteristics

A total of 278 patients (46% female) were included in the study and grouped according to their CA diagnostic status: CA positive (*n* = 60), CA negative (*n* = 100), and CA unknown (*n* = 118). The mean age at the time of CMR was significantly higher in the CA positive group (69.9 ± 10.3 years) compared with both the CA negative (59.7 ± 14.2 years) and CA unknown (62.9 ± 16.4 years) groups (*P* < 0.001). No significant differences were observed in gender, height, weight, body mass index, or body surface area between groups. Systolic blood pressure was significantly lower in CA positive patients (124.1 ± 20.3 mmHg) compared to CA negative (141.0 ± 27.6 mmHg) and CA unknown patients (135.1 ± 25.6 mmHg) (*P* < 0.001). Diastolic blood pressure and heart rate did not differ significantly across the groups (*P* = 0.08 and *P* = 0.89, respectively, Table 1).

**Table 1:** Baseline demographics and characteristics of patients at the time of CMR, and diagnostic criteria used for the diagnosis or exclusion of CA

Patient data	CA positive	CA negative	CA Unknown	Total	*P* value
Baseline characteristics					
Number of patients (n)	60	100	118	278	N/A
Gender (n, %) Male Female	39 (65%) 21 (35%)	53 (53%) 47 (47%)	58 (49%) 60 (51%)	150 (54%) 128 (46%)	0.13
Age at time of CMR (years)	69.9 ± 10.3	59.7 ± 14.2	62.9 ± 16.4	63.5 ± 14.9	< 0.001*^†^
Height (cm)	1.7 ± 0.1	1.7 ± 0.1	1.7 ± 0.1	1.7 ± 0.1	0.82
Weight (kg)	81.1 ± 19.2	87.5 ± 21.1	87.8 ± 20.7	84.5 ± 21.7	0.27
Body mass index (kg/m^2^)	27.6 ± 4.9	30.1 ± 7.2	29.0 ± 7.1	29.1 ± 6.8	0.09
Body surface area (m^2^)	1.9 ± 0.3	2.0 ± 0.3	1.9 ± 0.3	2.0 ± 0.3	0.56
Systolic blood pressure (mmHg)	124.1 ± 20.3	141.0 ± 27.6	135.1 ± 25.6	134.8 ± 26.0	< 0.001*^†^
Diastolic blood pressure (mmHg)	72.7 ± 11.8	78.6 ± 16.5	75.1 ± 15.9	75.8 ± 15.5	0.08
Heart rate (beats per minute)	73.6 ± 14.5	74.8 ± 15.8	72.9 ± 13.5	73.7 ± 14.6	0.89
**Diagnosis or exclusion of CA**					
Cardiac Biopsy 99 m Tc-PYP scan with serum immunofixation for SFLC AL amyloidosis with suspected CA No ‘gold standard’ test for CA performed *Clear alternative diagnosis to CA:* Hypertrophic Cardiomyopathy Non-ischemic dilated cardiomyopathy Hypertensive heart disease Cardiac Sarcoidosis Aortic Stenosis Other	17 (28%) 18 (30%) 25 (42%) 0 (0%) **0 (0%)** 0 (0%) 0 (0%) 0 (0%) 0 (0%) 0 (0%) 0 (0%)	24 (24%) 19 (19%) 0 (0%) 0 (0%) **57 (57%)** 24 (24%) 15 (15%) 7 (7%) 3 (3%) 2 (2%) 6 (6%)	0 (0%) 0 (0%) 0 (0%) 118 (100%) **0 (0%)** 0 (0%) 0 (0%) 0 (0%) 0 (0%) 0 (0%) 0 (0%)	41 (15%) 37 (13%) 25 (9%) 118 (42%) **57 (21%)** 24 (9%) 15 (5%) 7 (3%) 3 (1%) 2 (1%) 6 (2%)	**< 0.001**

Data represented as mean ± standard deviation. AL = amyloid light-chain; CA = cardiac amyloidosis; CMR = cardiac magnetic resonance; SFLC = serum free light chains; 99 m Tc-PYP = 99 m Technetium-pyrophosphate. * = statistically significant difference between CA positive and CA unknown; ^†^ = statistically significant difference between CA positive and CA negative. Significance indicated by bold font *p* < 0.05. ‘Clear alternative diagnosis to CA’ in bold font represents the sum of patients diagnosed with hypertrophic cardiomyopathy, non-ischemic dilated cardiomyopathy, hypertensive heart disease, cardiac sarcoidosis, aortic stenosis and other categories

### CA diagnosis

Among the 60 patients diagnosed with CA, 17 patients (28%) were confirmed by cardiac biopsy, 18 patients (30%) by 99mTc-PYP scintigraphy with negative serum immunofixation for serum free light chain analysis, and 25 patients (42%) had systemic AL amyloidosis with suspected cardiac involvement. In contrast, the CA negative group included 24% (*n* = 24) who had CA excluded by cardiac endomyocardial biopsy, 19 patients (19%) had CA excluded with a negative 99mTc-PYP scintigraphy with negative serum immunofixation for serum free light chain analysis, with no cases of AL amyloidosis. The remaining 57 patients (57%) had a clear alternative diagnosis made to explain the cause of the LVH including 24 patients with hypertrophic cardiomyopathy (42%); 15 patients with non-ischemic dilated cardiomyopathy (26%), 7 patients with hypertensive heart disease (12%), 3 patients with cardiac sarcoidosis (5%), 2 patients with LVH from significant aortic stenosis (4%) and 6 other (11%) which included 1 case of non-compaction cardiomyopathy, 2 cases of ischemic cardiomyopathy, 1 case of mitral valve prolapse, and 2 normal studies. In the CA unknown group, 118 patients had not received a gold-standard diagnostic test for CA nor had a clear diagnosis for their cause of LVH, accounting for 42% of the entire cohort. The distribution of diagnostic categories across the 3 groups was significantly different (*χ²* = 521.9, df = 20, *P* < 0.001, Table 1).

### CMR parameters

There were no differences between LV and RV volumes and ejection fraction when comparing the CA positive, CA negative, and CA unknown groups. A statistically significant difference was observed in LV cardiac output, which was lower in the CA positive group (4.9 ± 1.5 L/min) when compared to CA negative (5.3 ± 2.1 L/min) and CA unknown (5.7 ± 1.9 L/min) (*P* < 0.05). Significant differences were observed in native T1 values, which were elevated in CA positive patients (1122.4 ± 64.6 ms), compared with CA negative (1056.8 ± 69.7 ms) and CA unknown (1051.4 ± 54.0 ms) groups (*P* < 0.001). Similarly, post-contrast T1 was significantly lower in CA positive patients (364.6 ± 101.6 ms) compared with CA negative (427.7 ± 81.4 ms) and CA unknown (423.7 ± 86.4 ms) groups (*P* < 0.001). ECV was significantly elevated in CA positive patients (46.4 ± 11.5%) relative to CA negative (32.1 ± 7.2%) and CA unknown (31.4 ± 7.6%) groups (*P* < 0.001). Infarct-atypical LGE segments were significantly more common in CA positive patients (10.2 ± 7.3) compared with CA negative (2.7 ± 4.7) and CA unknown (2.0 ± 4.3) patients (*P* < 0.001; Table 2.

**Table 2:** CMR biventricular function and volumes, and tissue characterization parameters for patients with concentric LVH and a diagnosis of CA (CA positive), exclusion of CA (CA negative) or CA status unknown (CA unknown)

Patient data	CA positive	CA negative	CA Unknown	Total	*P* value
Ventricular volumes and function					
LV end diastolic volume (ml)	146.7 ± 41.3	175.6 ± 79.7	165.8 ± 65.7	165.8 ± 67.4	0.203
LV end diastolic volume indexed (ml/m^2^)	78.2 ± 21.2	88.1 ± 34.0	85.0 ± 30.4	84.6 ± 30.2	0.292
LV end systolic volume (ml)	79.7 ± 35.0	101.8 ± 76.7	86.3 ± 54.9	90.4 ± 61.0	0.229
LV end systolic volume indexed (ml/m^2^)	41.7 ± 18.9	50.5 ± 34.0	44.0 ± 25.6	45.9 ± 27.9	0.309
LV stroke volume (ml)	69.9 ± 25.9	73.8 ± 29.4	79.5 ± 28.6	75.4 ± 28.5	0.074
LV cardiac output (L/min)	4.9 ± 1.5	5.3 ± 2.1	5.7 ± 1.9	5.4 ± 1.9	< 0.05*
LV ejection fraction (%)	47.5 ± 13.3	45.7 ± 16.4	50.4 ± 13.3	48.1 ± 14.6	0.122
Myocardial mass diastole (g)	158.0 ± 51.2	160.7 ± 63.4	142.5 ± 53.6	152.4 ± 57.5	0.069
RV end diastolic volume (ml)	149.7 ± 41.3	175.6 ± 79.7	165.8 ± 65.7	165.8 ± 67.4	0.203
RV end diastolic volume indexed (ml/m^2^)	82.5 ± 24.4	82.8 ± 30.3	85.0 ± 28.3	84.0 ± 28.2	0.795
RV end systolic volume (ml)	79.7 ± 35.0	101.8 ± 76.7	86.3 ± 54.9	90. ± 61.0	0.229
RV end systolic volume indexed (ml/m^2^)	46.9 ± 20.8	48.7 ± 29.3	45.5 ± 21.2	47.0 ± 24.3	0.839
RV stroke volume (ml)	69.9 ± 25.9	73.8 ± 29.4	79.5 ± 28.6	75.4 ± 28.5	0.074
RV cardiac output (l/min)	4.8 ± 1.5	5.0 ± 2.1	5.5 ± 1.9	5.2 ± 1.9	0.066
RV ejection fraction (%)	44.1 ± 12.6	44.7 ± 15.8	47.6 ± 11.2	45.8 ± 13.4	0.160
LV tissue characterization					
Native T1 Mapping Global (ms)	1122.4 ± 64.6	1056.8 ± 69.7	1051.4 ± 54.0	1068.7 ± 68.3	< 0.001*^†^
Post Contrast T1 Mapping Global (ms)	364.6 ± 101.6	427.7 ± 81.4	423.7 ± 86.4	412.2 ± 9.4	< 0.001*^†^
ECV Global (%)	46.4 ± 11.5	32.1 ± 7.2	31.4 ± 7.6	35.3 ± 10.3	< 0.001*^†^
T2 Mapping Global (ms)	53.0 ± 7.4	50.8 ± 5.1	50.7 ± 3.9	51.3 ± 5.4	0.054
LGE Infarct-typical segments (mean n, SD)	0.4 ± 1.3	0.6 ± 1.6	0.5 ± 1.4	0.5 ± 1.4	0.654
LGE Infarct-atypical segments (mean n, SD)	10.2 ± 7.3	2.7 ± 4.7	2.0 ± 4.3	4.1 ± 6.1	< 0.001*^†^

CA = cardiac amyloidosis; ECV = extracellular volume; LGE = late gadolinium enhancement; LV = left ventricle; RV = right ventricle. * = statistically significant difference between CA positive and CA unknown; ^†^ = statistically significant difference between CA positive and CA negative. Significance indicated by bold font *p* < 0.05

Cine sequences suitable for 2D feature-tracking strain (FTS) post-processing were available in 50 CA-positive and 85 CA-negative patients. Basal peak systolic strain was significantly reduced in the CA positive group when compared to the CA negative group in the radial (18.8 ± 9.9% vs. 24.0 ± 10.8%, *p* < 0.005), circumferential (−12.7 ± 4.3% vs. −14.2 ± 4.8%, *p* < 0.05) and longitudinal (−11.6 ± 5.2% v −14.7 ± 4.8%, *p* < 0.005) dimensions, with no significant differences in mid-ventricular, apical and global peak systolic strain across all dimensions. Basal diastolic strain rate was significantly reduced in the CA positive group in the radial (−0.3 ± 0.7 s^− 1^ vs. −0.9 ± 0.1 s^− 1^, *p* < 0.005), circumferential (0.1 ± 0.8 s^− 1^ vs. 0.4 ± 0.8 s^− 1^, *p* < 0.01) and longitudinal (0.7 ± 0.4 s^− 1^ ± 0.9 ± 0.4 s^− 1^, *p* < 0.005) dimensions with no significant differences in mid-ventricular and apical diastolic strain rate across all dimensions. Global diastolic strain rates were significantly reduced in the radial (−0.1 ± 0.8 s^− 1^ vs. −0.4 ± 0.8 s^− 1^, *p* < 0.05) and circumferential (−0.1 ± 0.8 s^− 1^ vs. 0.3 ± 0.7 s^− 1^, *p* < 0.05) dimensions (Table 3).

**Table 3:** CMR feature tracking strain (FTS) parameters patients with concentric LVH and a diagnosis of CA (CA positive) or exclusion of CA (CA negative)

FTS parameter	Region	Dimension	CA positive (*n* = 50)	CA negative (*n* = 85)	*P* Value
Peak systolic strain (%)	Basal	Radial	18.8 ± 9.9	24.0 ± 10.8	**< 0.005**
		Circumferential	−12.7 ± 4.3	−14.2 ± 4.8	**< 0.05**
		Longitudinal	−11.6 ± 5.2	−14.7 ± 4.8	**< 0.005**
	Mid	Radial	16.4 ± 7.8	16.9 ± 8.8	0.95
		Circumferential	−14.5 ± 4.5	−13.6 ± 5.1	0.40
		Longitudinal	−6.9 ± 4.5	−8.0 ± 5.7	0.20
	Apical	Radial	22.6 ± 10.9	21.7 ± 11.9	0.57
		Circumferential	−16.5 ± 4.9	−15.7 ± 5.8	0.51
		Longitudinal	−10.6 ± 5.2	−10.2 ± 5.4	0.79
	Global	Radial	17.7 ± 8.2	19.4 ± 8.8	0.24
		Circumferential	−13.9 ± 4.1	−14.1 ± 4.9	0.57
		Longitudinal	−9.5 ± 4.4	−10.7 ± 4.1	0.06
Diastolic strain rate (s-1)	Basal	Radial	−0.3 ± 0.7	−0.9 ± 0.1	**< 0.005**
		Circumferential	0.1 ± 0.8	0.4 ± 0.8	**< 0.01**
		Longitudinal	0.7 ± 0.4	0.9 ± 0.4	**< 0.005**
	Mid	Radial	0.1 ± 1.1	−0.3 ± 0.8	< 0.05
		Circumferential	−0.2 ± 1.0	0.3 ± 0.8	< 0.01
		Longitudinal	0.5 ± 0.4	0.5 ± 0.5	0.19
	Apical	Radial	−0.2 ± 1.4	−0.3 ± 0.9	0.43
		Circumferential	0.1 ± 1.2	0.4 ± 0.9	0.09
		Longitudinal	0.7 ± 0.4	0.6 ± 0.5	0.43
	Global	Radial	−0.1 ± 0.8	−0.4 ± 0.8	**< 0.05**
		Circumferential	−0.1 ± 0.8	0.3 ± 0.7	**< 0.05**
		Longitudinal	0.5 ± 0.2	0.5 ± 0.3	0.16

CA = cardiac amyloidosis. *P* < 0.05 in bold font indicates significance

### ROC analysis

**Fig. 1:**
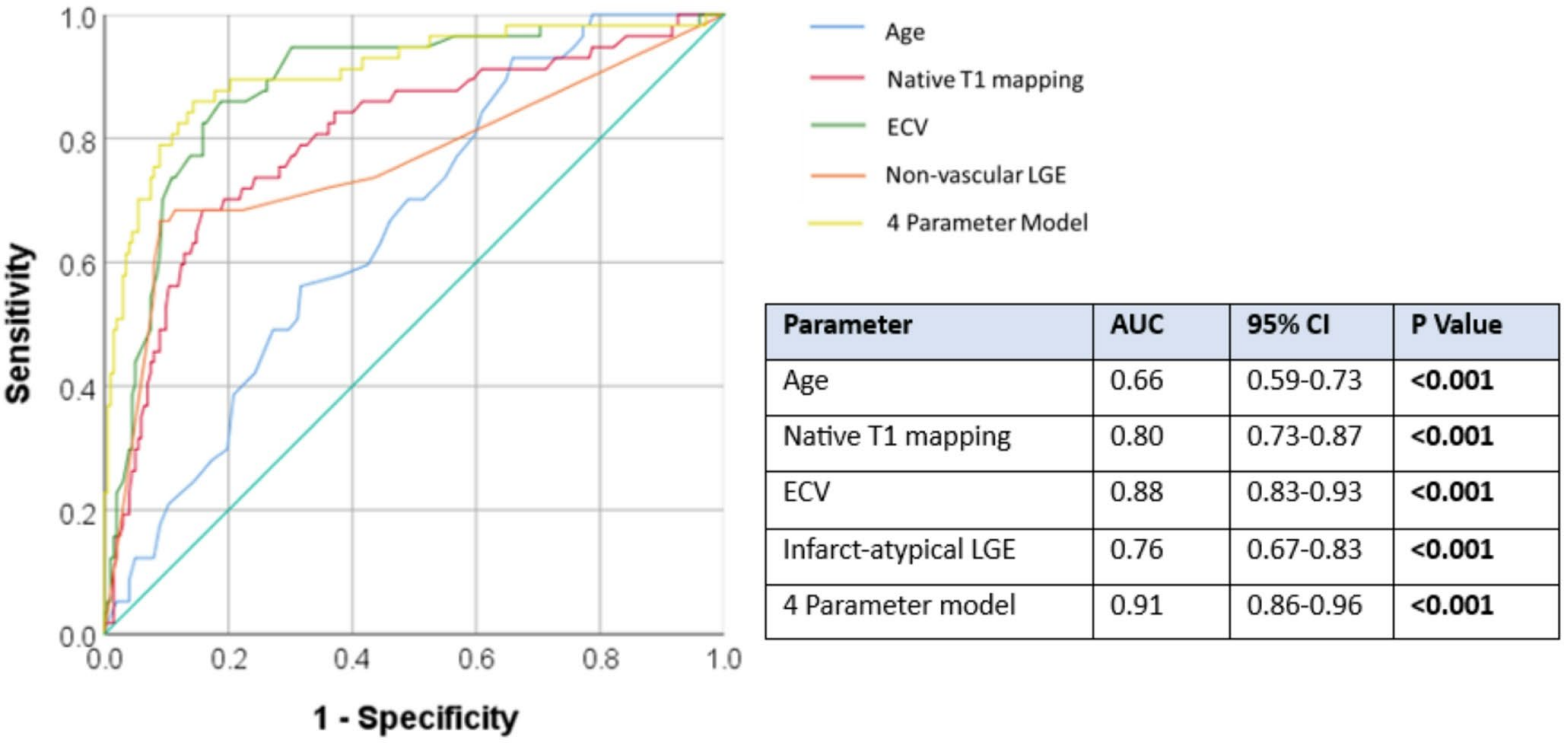
Multiparametric CMR ROC curves for diagnosis of CA when compared CA negative or undifferentiated concentric LVH patients. A 4 parameter model including age, native T1 mapping, ECV, number of segments with infarct-atypical LGE determined an AUC of 0.91 for detection of CA. AUC 0.91 (CI 0.86–0.96) *P* < 0.001. Individual AUCs for native T1 mapping (red), ECV (green), infarct-atypical LGE (orange), and age (blue) are presented in the accompanying table. AUC = area under curve, ECV = extracellular volume, LGE = late gadolinium enhancement, ROC = receiver operator characteristics

**Fig. 2:**
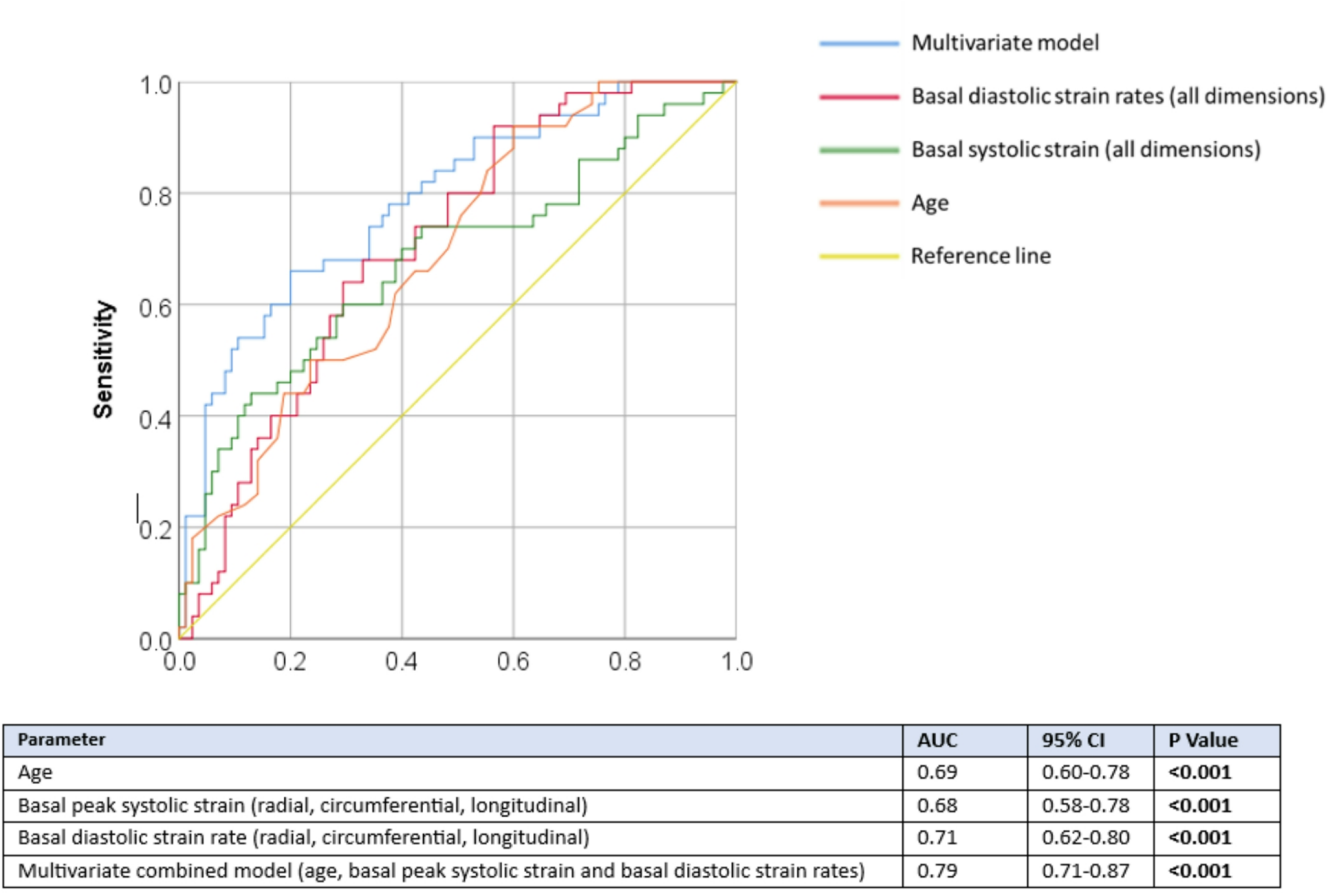
Multiparametric CMR ROC curves for diagnosis of CA when compared CA negative patients. A 3 parameter model including age, basal peak systolic strain and basal diastolic strain rate demonstrated an of AUC 0.79 (CI 0.71–0.87) *P* < 0.001 for discrimination of CA. Individual AUCs basal diastolic strain rate (red), basal systolic strain (green) and age (orange), are presented in the accompanying table. AUC = area under curve, ROC = receiver operator characteristics

ROC curve analysis was performed to evaluate the diagnostic performance of individual imaging markers and a combined model for the detection of CA. Among the individual parameters, ECV demonstrated the highest discriminatory power, with an area under the curve (AUC) of 0.88 (95% CI: 0.83–0.93, *P* < 0.001) and sensitivity of 82% and specificity of 83%, using an optimal cut-off ECV value of 37%. This was followed by native T1 mapping, which achieved an AUC of 0.80 (95% CI: 0.73–0.87, *P* < 0.001) with sensitivity of 71% and specificity of 74% using an optimal cut-off value of 1093 ms. Infarct-atypical late gadolinium enhancement (LGE) demonstrated an AUC of 0.76 (95% CI: 0.67–0.83, *P* < 0.001), with a sensitivity of 73% and specificity of 63%, using a cut-off of at least 2 myocardial segments containing infarct-atypical LGE. Age alone was a less accurate predictor of CA, with an AUC of 0.66 (95% CI: 0.59–0.73, *P* < 0.001), indicating limited discriminative value as a standalone predictor, with sensitivity of 92% and specificity of 38% using a cut-off age of ≥ 58 years. A multivariable logistic regression model incorporating 4 parameters: age, native T1 mapping, ECV, and infarct-atypical LGE, yielded an AUC of 0.91 (95% CI: 0.86–0.96, *P* < 0.001) with an overall accuracy of 83.9% in detecting CA (Fig. 1). This logistic regression model was applied to 118 CA unknown patients to estimate the probability of CA. A probability threshold of ≤ 0.5 was used to classify patients as likely to have CA. Of this group 13 patients (11%) were deemed as likely to have CA (Fig. 2).

## Discussion

The aim of this study was to evaluate the diagnostic utility of CMR, including both quantitative and qualitative tissue characterization parameters, in distinguishing CA from other causes of concentric LVH. Importantly, this retrospective study was conducted in a real-world cohort of patients with undifferentiated LVH, reflective of the diagnostic uncertainty frequently encountered in clinical practice when identifying CA with CMR. In these patients undergoing CMR to determine etiology of LVH, we found that ECV, native T1 mapping, and infarct-atypical LGE provided strong discriminatory power for detecting CA, with ECV showing the highest individual diagnostic performance. Our findings further demonstrate that a multivariable model incorporating age, ECV, native T1, and LGE significantly improves diagnostic accuracy for CA, achieving an AUC of 0.91.

ECV emerged as the strongest individual CMR parameter (AUC = 0.88) with a sensitivity of 82% and specificity of 83% using a cut-off value of 37% in our study, consistent with prior studies reporting its utility in detection of CA. It has been identified as an early marker for myocardial burden of amyloid protein [4, 13–15], and ECV in CA is at significantly higher levels than other cardiomyopathies [16]. In the correct clinical context, ECV > 40% has been reported to be consistent with a CA diagnosis [17]. ECV has been associated with prognosis in both AL and ATTR amyloidosis [14, 18, 19], and a decrease in ECV has been associated with response to therapy in amyloidosis [20, 21]. Our study further supports the utility of ECV in discrimination of CA from other cardiomyopathies.

Myocardial LGE has demonstrated high specificity and moderate sensitivity in detecting cardiac amyloidosis in endomyocardial biopsy-proven CA [22, 23] and furthermore ATTR can be differentiated from AL type CA with 96% specificity and 87% sensitivity with ‘Query Amyloid Late Enhancement’ (QALE) scoring [24]. LGE is usually distributed in a non-coronary subendocardial pattern with associated abnormal blood pool kinetics [25–27], which in time may progress to transmural left and right ventricular myocardial enhancement [14, 15, 27]. Abnormal blood pool kinetics with myocardial nulling either at the same time or before the blood pool inversion time may be evident. Indeed, identification of transmural LGE has been associated with poor prognosis in CA reflecting significant amyloid burden [28–30]. LGE however is not a validated quantitative measure in CA and thus lacks the potential to track response to therapies over time. Cases of suboptimal myocardial nulling despite good technique due to abnormal blood pool kinetics [30], as well as cases where LGE does not occur or atypical patchy focal distribution of LGE in cardiac amyloidosis have also been identified, even in patients with severe disease [23, 28] which could potentially impact appropriate image interpretation [30]. It is likely that where there is diffuse infiltrative LGE distributions that occur in CA, it is difficult to identify ‘remote’ or normal myocardium as a reference for establishing signal-intensity thresholds for quantitative LGE measures. In our study, LGE showed lower discriminative power (AUC = 0.76) than ECV. The sensitivity and specificity were modest at 73% and 63% respectively, likely due to overlap with other infiltrative and other fibrotic pathologies in the CA negative and CA unknown groups. Abnormal blood pool kinetics, a categorical finding, was not suitable for quantitative diagnostic threshold determination and therefore not included in the regression model. Nonetheless, the additive diagnostic value of combining LGE with native T1 and ECV was evident in the multivariate model which overall demonstrated high discriminatory power with an AUC of 0.91.

Native T1 mapping also demonstrated good diagnostic performance in this study, supporting its role as a non-contrast alternative for CA assessment, particularly in patients with contraindications to gadolinium. Native T1 mapping allows for quantitative measurement of signal from the myocardium, and has the potential to detect early changes in myocardial tissue characteristics even before the development of LGE [4, 15, 28, 31, 32]. Increased T1 correlates with increased LV thickening and reduced LV systolic function or increased diastolic dysfunction in CA [32]. Previous studies have demonstrated significantly elevated native T1 in patients with CA vs. both non-amyloid hypertrophic cardiomyopathies, aortic stenosis with comparable myocardial hypertrophy and normal controls [13, 28, 32, 33], and high native T1 mapping has been linked to a poor prognosis in CA [18, 30]. The potential diagnostic value of T1 mapping in CA was described by White et al. in 2014 [30], who evaluated the diagnostic and prognostic use of a rapid non-quantitative visual T1 assessment in a ‘real-world’ population undergoing CMR for suspected CA. With this approach it was determined in a subgroup of 25 patients with cardiac pathology validation that diffuse hyperenhancement determined by visual T1 assessment demonstrated a sensitivity and specificity in the diagnosis of CA of 93% and 70% in this study sample, respectively. More recently, Baggiano et al. [34] performed a prospective study examining the diagnostic use of T1 mapping to accurately diagnose CA in a sample group with 50.8% prevalence of CA. Native T1 mapping if myocardium showed high diagnostic accuracy for diagnosis of CA with an area under curve of 0.93. However despite this potential for CA diagnosis, native T1 values vary due to many factors including field strength, MRI vendor, as well as patient factors including age, gender and heart-rate variability. We determined an AUC of 0.80 for detection CA, with an optimal cut-off of 1093ms yielding a sensitivity of 71% and specificity of 74%, which supports prior studies demonstrating the utility of native T1 in detection of CA.

In regards to FTS, we observed selective basal myocardial dysfunction in patients with CA, as demonstrated by significantly impaired basal peak systolic strain and reduced basal diastolic strain rates in all dimensions (radial, circumferential and longitudinal). The “apical sparing” pattern, particularly in longitudinal strain, has been widely reported as a hallmark of CA [35]. These findings, showed low to modest discriminatory power for detection of CA when compared to a heterogenous group of LVH patients. This likely reflects the overlap in strain abnormalities seen in other causes of LVH which can also exhibit regional strain abnormalities [36]. Nevertheless, basal strain impairment may hold diagnostic value when integrated with other imaging parameters and clinical factors, warranting further investigation in future studies.

**Fig. 3:**
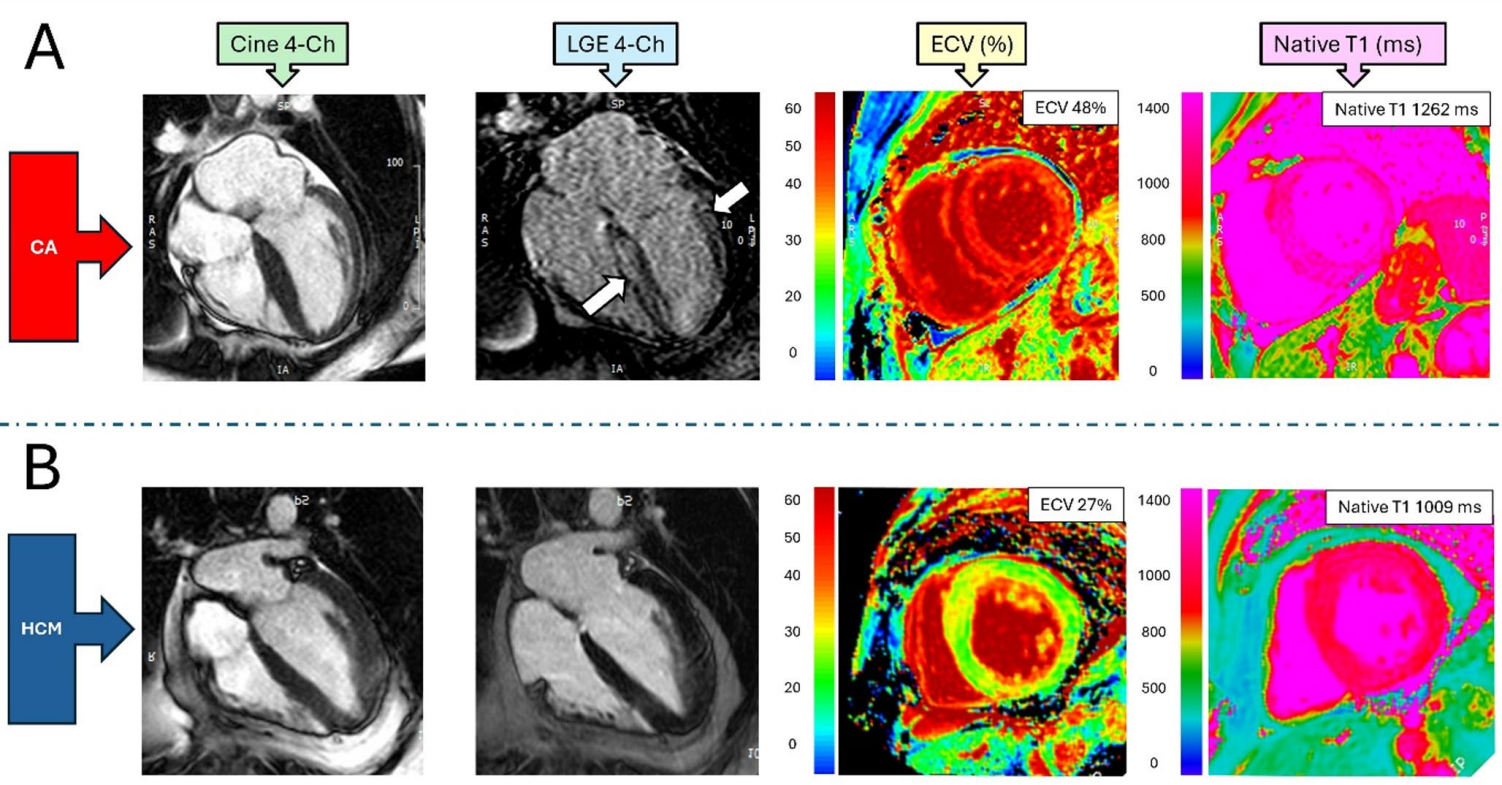
Representative patients who underwent multiparametric tissue characterization for undifferentiated LV hypertrophy. Patient (**A**) is 57 year old female. LGE sequences demonstrated diffuse subendocardial to midmyocardial delayed enhancement (white arrows) with an elevated ECV of 48% and elevated native T1 of 1262 ms at 1.5T cardiac MRI. This patient was subsequently diagnosed with AL amyloidosis. Patient (**B**) is a 53 year old male, LGE sequences demonstrated RV insertion point LGE (not shown) and a normal ECV of 27% and native T1 of 1009 ms. This patient was subsequently diagnosed with HCM. AL = amyloid light chain; 4-Ch = 4-chamber; CA = cardiac amyloidosis; ECV = extracellular volume fraction; HCM = hypertrophic cardiomyopathy; LGE = late gadolinium enhancement; LV = left ventricle; RV = right ventricle

A 4 parameter logistic regression model consisting of age, native T1, infarct-atypical LGE and ECV yielded the highest discrimination for detection of CA with an AUC of 0.91 and sensitivity of 92% and specificity of 81%. When this logistic regression prediction model was applied to the CA unknown group, 13 patients (11% of the CA unknown group) were deemed likely to have CA. Overall, this studies findings suggest that predictive multiparameter models derived from CMR may aid in the detection of CA. Such models could represent a useful adjunct to conventional diagnostic approaches for CA (Fig. 3).

## Limitations

The diagnosis of CA in this study was established using contemporary criteria, including cardiac biopsy, 99mTc-PYP scintigraphy, and evidence of systemic amyloidosis; however, not all patients underwent gold-standard testing. The CA-unknown group, comprising 42% of the cohort, underscores the diagnostic ambiguity that often arises in real-world settings, where comprehensive evaluation—such as endomyocardial biopsy or full systemic workup—is frequently incomplete or unavailable. This group may include individuals with early or subclinical CA not yet meeting diagnostic thresholds, as well as patients with non-amyloid causes of LVH. The majority of the ‘CA unknown’ group had a documented history of hypertension, which would be suggestive as a probable cause of LVH in the majority of this subgroup. Additionally, FTS analysis was only feasible in a subset of patients, reflecting heterogeneity in imaging protocols and post-processing software compatibility across the cohort. Myocardial strain was therefore assessed separately as including FTS in the primary multivariable prediction model would have reduced the sample size and potentially introduced selection bias. We therefore chose to present FTS analyses independently to provide additional mechanistic and functional context while maintaining the integrity and statistical robustness of the primary model. Generalizability of these findings would require further validation in larger, multi-centre studies using multi-vendor imaging platforms.

## Conclusion

In a heterogeneous clinical cohort undergoing CMR for LVH assessment, ECV, native T1 mapping, and infarct-atypical LGE were independently associated with a diagnosis of CA. A multivariable model incorporating age and imaging biomarkers significantly improved diagnostic accuracy, suggesting a potential role for multiparametric CMR in the detection of CA. These findings support the routine integration of parametric mapping and tissue characterization into CMR protocols for undifferentiated LVH.

## Human ethics and consent

## Data Availability

No datasets were generated or analysed during the current study.
